# No sex-related differences in PANSS score reductions in adult patients with acutely exacerbated schizophrenia treated with Risperidone ISM

**DOI:** 10.1192/j.eurpsy.2025.894

**Published:** 2025-08-26

**Authors:** C. Sherifi, J. Martínez González, L. Anta Carabias, M. Almendros Gimenez, C. Salazar García, C. U. Correll

**Affiliations:** 1 Medical Affairs, ROVI Biotech Ltd., Croydon, United Kingdom; 2 Medical Department, Laboratorios Farmaceuticos ROVI S.A., Madrid, Spain; 3 Department of Psychiatry and Molecular Medicine, Donald and Barbara Zucker School of Medicine at Hofstra/Northwell, Hempstead, NY; 4 Department of Psychiatry Research, The Zucker Hillside Hospital, Glen Oaks, NY, United States; 5 Department of Child and Adolescent Psychiatry, Charité Universitätsmedizin Berlin, Berlin, Germany; 6 Center for Psychiatric Neuroscience, The Feinstein Institute for Medical Research, New Hyde Park, NY, United States; 7 German Center for Mental Health, Partner Site Berlin, DZPG, Berlin, Germany

## Abstract

**Introduction:**

There is a call to consider sex differences in mental health research [Galbally *et al.* CNS Drugs 2024; 38(7):559-570; Ercis *et al.* J Affect Disord. 2024; 352:171-192]. Sex-related differences in risperidone efficacy have been reported to be limited [Galbally *et al.* CNS Drugs 2024; 38(7):559-570]. Risperidone ISM (Risp-ISM) is a monthly long-acting injectable (LAI) formulation of risperidone, recently authorised in Europe, USA and some other countries.

**Objectives:**

To investigate potential sex-related differences in the short-term efficacy of Risp-ISM LAI in adults with schizophrenia [Correll *et al.* NPJ Schizophr. 2020; 6(1):37].

**Methods:**

Post-hoc analysis of a double-blind (DB), randomised, placebo-controlled, 12-week study conducted in participants with acutely exacerbated schizophrenia (NCT03160521). Data from the Positive and Negative Syndrome Scale (PANSS) were analysed by sex to reveal potential differences in efficacy versus placebo. The data were analysed within three separate study groups: 75 mg Risp-ISM, 100 mg Risp-ISM and placebo using a mixed effect with repeated measures model (MMRM). Herein, PANSS total scores changes from baseline (the primary efficacy endpoint) are shown.

**Results:**

In the double-blind phase, 437 eligible participants were randomly assigned 1:1:1 to receive Risp-ISM 75 mg, 100 mg or placebo every 28 days. 144 (33%) were female and 293 (67%) male. Analysis showed no sex-related differences on PANSS total scores. After 12 weeks of treatment, the scores in PANSS Total as well in the Positive, Negative and General Psychopathology subscales were statistically significant lower for Risp-ISM 100 mg and 75 mg versus Placebo in both male and female subgroups. Specifically, decreases from baseline were significantly greater versus placebo at Day 8 (after first injection) and beyond in both sex subgroups at the 100 mg Risp-ISM dose versus Placebo; likewise, at Day 15 and beyond for the 75 mg Risp-ISM dose (Figures 1 and 2).

**Image 1:**

**Figure:**
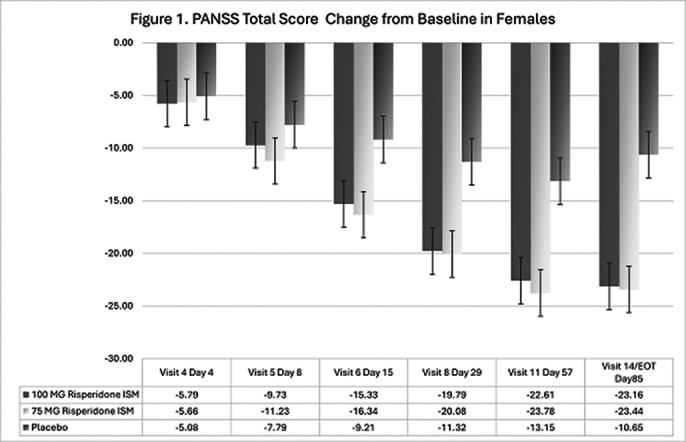

**Image 2:**

**Figure:**
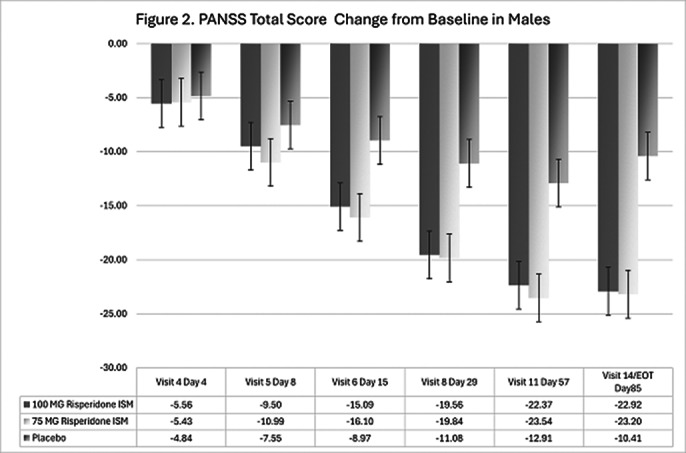

**Conclusions:**

There were no statistically significant differences in efficacy, measured as PANSS score change from baseline, for male or female participants versus placebo regardless of Risp-ISM dose. Risp-ISM LAI provided significant improvement of the symptomatology as early as 8 days after first injection in acutely exacerbated patients with schizophrenia regardless of their sex.

**Disclosure of Interest:**

C. Sherifi Employee of: ROVI Biotech Ltd., J. Martínez González Employee of: Laboratorios Farmaceuticos ROVI S.A., L. Anta Carabias Employee of: Laboratorios Farmaceuticos ROVI S.A., M. Almendros Gimenez Employee of: Laboratorios Farmaceuticos ROVI S.A., C. Salazar García Employee of: Laboratorios Farmaceuticos ROVI S.A., C. Correll Shareolder of: Cardio Diagnostics, Kuleon Biosciences, LB Pharma, Mindpax, and Quantic., Grant / Research support from: Janssen and Takeda., Consultant of: AbbVie, Acadia, Alkermes, Allergan, Angelini, Aristo, Biogen, Boehringer-Ingelheim, Cardio Diagnostics, Cerevel, CNX Therapeutics, Compass Pathways, Darnitsa, Denovo, Gedeon Richter, Hikma, Holmusk, IntraCellular Therapies, Jamjoom Pharma, Janssen/J&J, Karuna, LB Pharma, Lundbeck, MedAvante-ProPhase, MedInCell, Merck, Mindpax, Mitsubishi Tanabe Pharma, Mylan, Neurocrine, Neurelis, Newron, Noven, Novo Nordisk, Otsuka, Pharmabrain, PPD Biotech, Recordati, Relmada, Reviva, Rovi, Sage, Seqirus, SK Life Science, Sumitomo Pharma America, Sunovion, Sun Pharma, Supernus, Takeda, Teva, Tolmar, Vertex, and Viatris.

